# Distribution and Sources of Nitrate-Nitrogen in Kansas Groundwater

**DOI:** 10.1100/tsw.2001.331

**Published:** 2001-11-09

**Authors:** Margaret A. Townsend, Stephen A. Macko, David P. Young

**Affiliations:** Kansas Geological Survey, Lawrence 66047, USA

**Keywords:** nitrate, nitrate-N, groundwater, nitrogen isotopes, statistics, Kansas, water quality, agriculture, irrigation

## Abstract

Kansas is primarily an agricultural state. Irrigation water and fertilizer use data show long- term increasing trends. Similarly, nitrate-N concentrations in groundwater show long-term increases and exceed the drinking-water standard of 10 mg/l in many areas. A statistical analysis of nitrate-N data collected for local and regional studies in Kansas from 1990 to 1998 (747 samples) found significant relationships between nitrate-N concentration with depth, age, and geographic location of wells. Sources of nitrate-N have been identified for 297 water samples by using nitrogen stable isotopes. Of these samples, 48% showed fertilizer sources (+2 to +8) and 34% showed either animal waste sources (+10 to +15 with nitrate-N greater than 10 mg/l) or indication that enrichment processes had occurred (+10 or above with variable nitrate-N) or both. Ultimate sources for nitrate include nonpoint sources associated with past farming and fertilization practices, and point sources such as animal feed lots, septic systems, and commercial fertilizer storage units. Detection of nitrate from various sources in aquifers of different depths in geographically varied areas of the state indicates that nonpoint and point sources currently impact and will continue to impact groundwater under current land uses.

## INTRODUCTION

Groundwater is the major source of drinking water for 50% of the residents in Kansas[1]. Nitrate-N is one of the most widespread inorganic contaminants in Kansas groundwater. The drinking-water limit above which nitrate is considered a health risk is 10 mg/l as nitrogen[2]. In this paper, the term nitrate is used to denote the nitrate-N content of the water.

Background level for the natural occurrence of nitrate in pristine areas of the U.S. is considered less than or equal to 2 mg/l[3,4]. The objectives of this paper are to evaluate the potential sources of nitrate in groundwater in Kansas by use of nitrogen isotopes and to statistically analyze statewide nitrate data for the period of 1990 to 1998.

## METHODS

The primary sources of data consist of studies done by the Kansas Geological Survey (KGS) and by other agencies for which the KGS performed groundwater chemistry analyses. Water-well completion forms on file at the KGS, the Water Information Management and Analysis System (WIMAS) database[5], and field notes were used to obtain additional information concerning well construction. Water samples were collected and analyzed using standard field and laboratory methods, as reported elsewhere[6].

The nitrogen-15 analysis combusted samples of the dried nitrate-containing salts at high temperature to nitrogen gas for analysis of their isotopic compositions. The $\delta^{15}N$ was determined on a Micromass Optima isotope ratio mass spectrometer (IRMS) coupled with an elemental analyzer (EA), with an overall precision better than 0.5‰ (per mil). Results are reported relative to a standard (atmospheric $N_2$) defined to be 0‰ and expressed in standard $\delta$ notation[7].

Statistical analysis was performed with the SAS statistical package for UNIX systems[8]. Because of the lack of normality in the data set (Shapiro-Wilks probability test values for all data groups were less than $\alpha = 0.10$) nonparametric methods were used. The specific tests used were the Kruskal-Wallis test (analysis of variance equivalent), the Mann-Whitney *t*-test equivalent, and the Spearman rho test for correlation. All tests were performed at the 90% level of confidence ($\alpha = 0.10$)[6]. Factors with probability values greater than $\alpha = 0.10$ are not reported.

## BACKGROUND

Agriculture is the dominant industry in Kansas. The major agricultural commodities for the year 2000 include corn for grain, soybeans, sorghum for grain, wheat, cattle, dairy, and hogs[9]. Because of the extensive agriculture in the state, many of the sources of nitrate are related to farming activities but are considered nonpoint sources.

Rainfall across the state is variable (approximately 40.5 cm/year in western Kansas to over 100 cm/year in eastern Kansas). Irrigated agriculture, particularly in the western two-thirds of Kansas, has increased since the 1940s, as illustrated by the cumulative irrigation water rights and the volume of nitrogen fertilizer sold statewide[9] (Fig. 1).

Animal waste (including human waste) is another potential point source of nitrate (feedlots, septic systems, and sewage waste-treatment systems) and nonpoint source (use of manure as fertilizer). The potential contribution of animal-waste nitrogen loading to the hydrologic system in Kansas is approximately 180,000 metric tons/year[9,10] if the waste is not properly utilized. The total amount of organic nitrogen from animal and human sources is less than 20% of the amount of nitrogen applied in the form of commercial fertilizers (900,000 metric tons, Fig. 1) in the year 2000.

Long-term agriculture has affected groundwater quality in other parts of the U.S.[11,12], resulting in increased nitrate concentrations. In many areas of Kansas, nitrate concentrations are above the background level of 2 mg/l observed in undeveloped regions in the U.S., indicating an anthropogenic impact on groundwater quality[3,6]. A set of irrigation wells in Kansas that was sampled for water chemistry in both the 1970s and the 1990s illustrates a long-term effect of irrigated agriculture — an over all increase in nitrate concentration (Fig. 2).

## IDENTIFICATION OF NITROGEN SOURCES

Natural abundance nitrogen isotope analysis is a method frequently used to assist in determining sources of nitrogen in groundwater. The isotope analysis involves establishing the ratio of nitrogen-15 ($^{15}N$) to nitrogen-14 ($^{14}N$) on the nitrogen in nitrate compared to the ratio observed in the standard, atmospheric nitrogen (air). Comparisons of these values indicate if there is more (positive) or less (negative) $^{15}N$ in the sample. The values thus indicate whether the sample is enriched (+) or depleted (–) in relation to the standard.

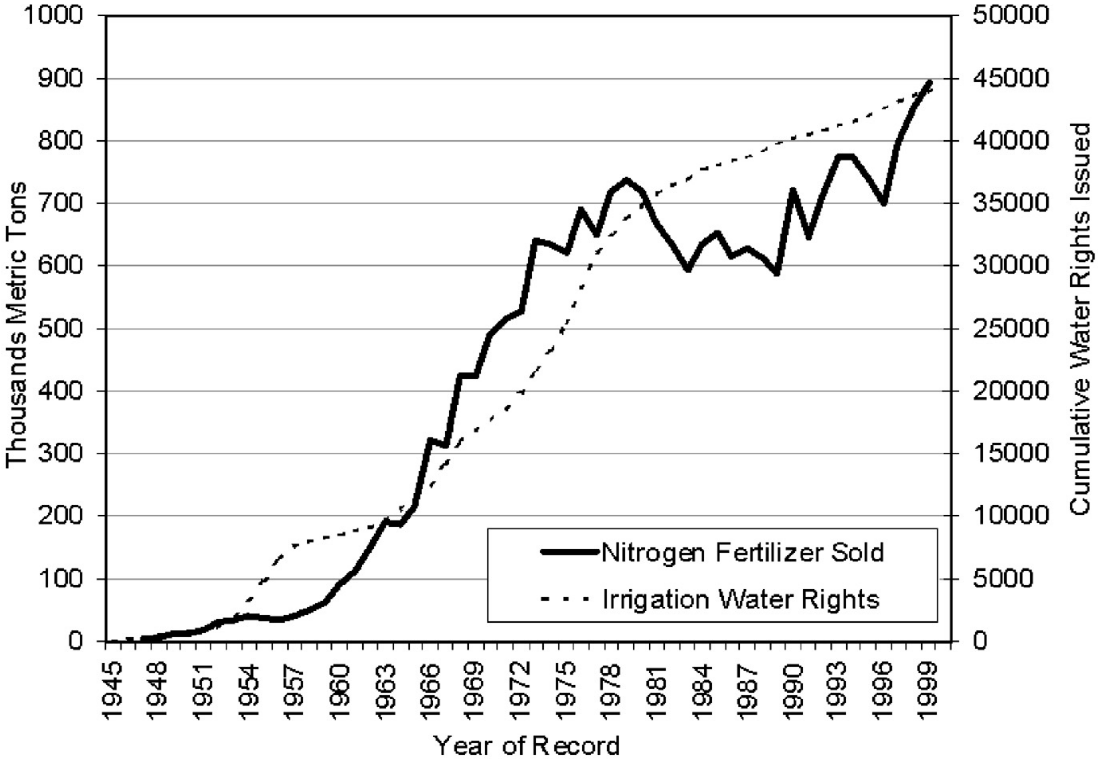

**FIGURE 1.** Cumulative irrigation water rights issued and quantity of nitrogen fertilizer sold in Kansas[5,9].

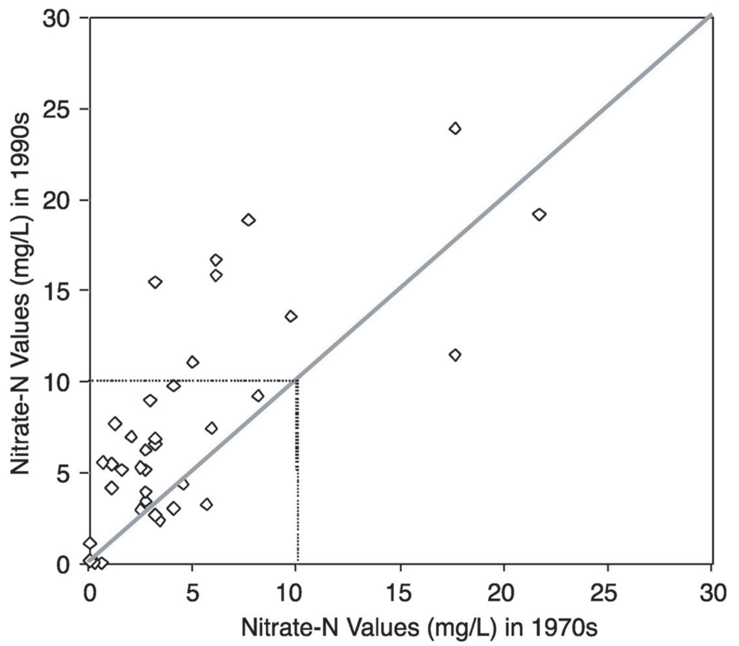

**FIGURE 2.** Measured nitrate values from samples collected from the same wells in 1970s and 1990s. Diagonal line indicates no change between two time periods. Box indicates nitrate-N drinking-water limit of 10 mg/l[2,13].

Isotopic values are reported as $\delta^{15}N$ in per mil (‰)[7]:

$$\delta^{15}N\ (‰) = \frac{(^{15}N/^{14}N)_{sample} - (^{15}N/^{14}N)_{standard}}{(^{15}N/^{14}N)_{standard}} \times 1000 \qquad (1)$$

Fig. 3 illustrates the range of $\delta^{15}N$ values for various sources of nitrogen and associated processes affecting the $^{15}N$ abundance[14]. Generally, biological activities use $^{14}N$ preferentially, resulting in an increased $\delta^{15}N$ value in the remaining substrate. Previous work has shown that nitrate from commercial fertilizer sources has $\delta^{15}N$ values of –2 to +8‰, from soil nitrogen a range of +5 to +7‰, and from animal waste generally greater than +10‰[14,15]. Other information, such as dissolved oxygen, salinity, iron, and manganese concentrations, and proximity to potential sources, aids in source determination.

Denitrification, the process of breaking down nitrate by bacteria to form molecular nitrogen gas, also results in a $\delta^{15}N$ range of +10 to greater than +20‰ in groundwater nitrate. The concentration of nitrate in groundwater is a strong indicator of whether isotopically altered animal waste or denitrification is the cause of an observed high $\delta^{15}N$ value. Animal-waste sources generally result in relatively high nitrate concentrations and denitrification processes generally result in relatively low nitrate concentrations.

Volatilization of nitrogen to ammonia gas can occur throughout the nitrogen cycle at pH > 8. The result of volatilization is an increased $\delta^{15}N$ value in the remaining nitrate but a variable nitrate concentration. Most volatilization effects in Kansas are seen in areas with silt loam or loess soils with observable caliche zones[16,17]. Research in western Nebraska has also illustrated the increase in $\delta^{15}N$ values when caliche zones cause changes in the water chemistry[14].

A total of 297 samples from site studies throughout Kansas were evaluated using nitrogen isotopes. Of these samples, 48% fall within the fertilizer category, 18% have mixed sources, and 34% have $\delta^{15}N$ values greater than +10‰ (Fig. 4). The majority of the wells sampled are domestic wells, with some irrigation, public water supply, and monitoring wells included. A site inspection of each location provided information concerning possible sources for nitrate.

For the purposes of this paper, the samples are identified by the area of the state where collection occurred (Fig. 5, index map). Area 1 is in western Kansas and consists of the deep Pliocene and Pleistocene High Plains aquifer overlain by thick loess deposits, caliche zones, and a deep water table of greater than 35 m. Area 2, in south-central Kansas, consists of shallow Pleistocene High Plains sediments, with areas of very sandy surface soils and vadose zone, and a shallow water table of 10 m or less. Irrigated farming is common in both Areas 1 and 2. Area 3, in north-central Kansas, consists generally of alluvial sediments along major rivers in this part of the state. Sediments range from silt loam with calcareous zones to sandy loams with clay lenses. Depth to water in this area is generally less than 5 m.

Of the 100 samples with $^{15}N$ enrichment above +10‰, 31 samples have nitrate values of less than 4 mg/l. These particular samples are indicative of alteration resulting in $^{15}N$ enrichment, either because of denitrification in a reducing groundwater environment and/or ammonia volatilization at the surface or en route

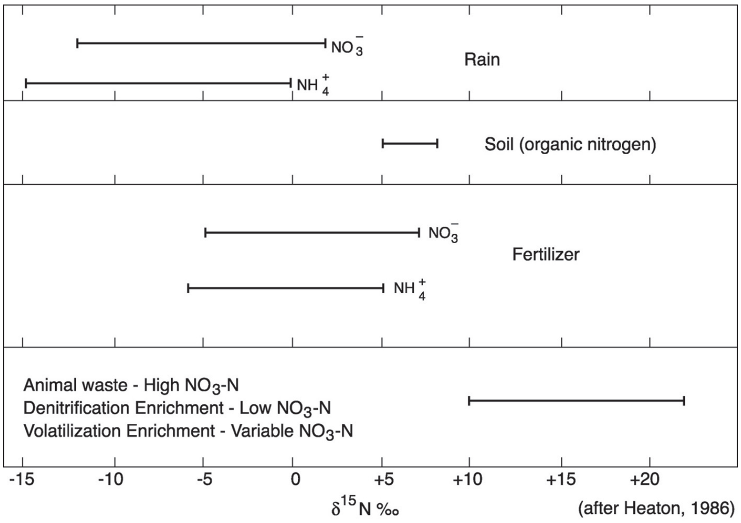

**FIGURE 3.** Range of $\delta^{15}N$ values for various sources of nitrogen and the enrichment effects of denitrification and volatilization processes[14].

through a thick vadose zone. The majority of these samples occur in the deep aquifers in western Kansas (Area 1, Figs. 4 and 5) or in areas with low permeability or calcareous rich soils in the vadose zone (Areas 1 and 3, Figs. 4 and 5)[16,17].

Of the samples with $^{15}N$ enrichment above +10‰, 49 have nitrate greater than 10 mg/l and are indicative of animal-waste sources. The samples in the animal-waste type water area of Fig. 4 are from feedlot or septic tank point sources as identified in site studies. These sites generally occurred in Areas 2 and 3. Current work indicates a relationship between increasing nitrate and chloride concentration in samples from alluvial areas, further supporting the animal-waste source interpretation (Area 3, Fig. 5). Evaluation of this relationship in the other two areas is difficult because of impacts by natural salinity sources.

The majority of samples that occur below the $\delta^{15}N$ line of +8‰ (fertilizer water-type) are from areas with irrigated farming, sandy soils, and no obvious animal-waste sources [6,13,16,17]. These samples represent the impact of nonpoint source farming on groundwaters of the state.

## FACTORS AFFECTING OCCURRENCE OF NITRATE IN KANSAS GROUNDWATER

Other studies throughout the U.S. have indicated that many factors can influence the occurrence of nitrate in groundwater[6,11,12]. These factors include depth of well, age of well, permeability of soils, depth to groundwater, and well construction practices, to name a few[18,19,20,21]. In Kansas, the major factors that are statistically correlated with the occurrence of nitrate in groundwater statewide include age of well, depth of well, and geographic location within the state[6,13]. Fig. 5 shows the location and distribution of nitrate of the 747 samples used in the statewide assessment.

### Geographic Area

Fig. 5 (index map) also shows the geographic subdivision of the state based primarily on aquifer depth, soil type, precipitation, and land use. The division of the state was based in part on data available from previous water-quality studies within the state[6,13,16,17]. Area 1 in western Kansas consists primarily of the deep High Plains aquifer with a thick vadose zone and a high density of irrigated farming. Area 2 in south-central Kansas is a portion of the High Plains aquifer with less saturated thickness, a thin vadose zone, sandier soils, and also a high density of irrigated farming. Area 3 consists of the alluvial systems sampled in north-central and eastern Kansas. The alluvial systems are generally the shallowest ground-water systems with variable sand, gravel, and clay zones and thin vadose zones.

The Kruskal-Wallis test shows a significant statistical difference between the geographic location of the samples and the observed nitrate concentrations, with the highest concentrations occurring in the alluvial systems (Area 3), intermediate concentrations in the south-central area (Area 2), and the lowest concentrations in western areas (Area 1; Table 1). Factors contributing to the observed nitrate values are well depth (shallower from west to east), soil type (sandier in Area 2), and thickness of vadose zone (thins from west to east with the thinnest zones occurring in alluvial systems). In addition, lower nitrate values in western Kansas may be related to biological reduction in the vadose zone in perched water occurring above caliche zones, as observed in Nebraska and in site studies in Kansas[15,16,17].

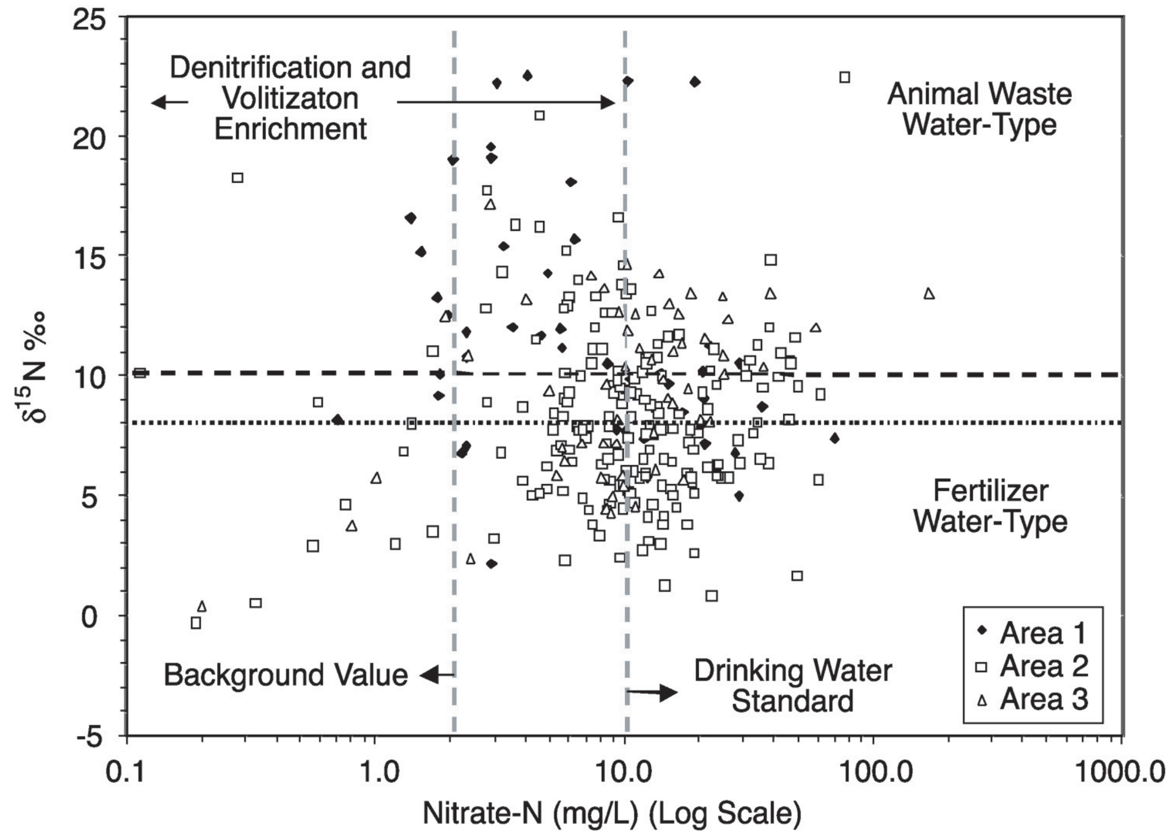

**FIGURE 4.** Summary diagram of potential sources and processes affecting nitrate and $\delta^{15}N$ values from site studies in Kansas[6,16,17]. Sources include fertilizer, animal waste, and soil nitrogen. Processes include denitrification or ammonia volatilization enrichment. Background nitrate value and drinking water standard taken from the literature[2,3,4]. Areas 1, 2, and 3 illustrated in Fig. 5 (index map).

## Age of Well

Poor well construction is often cited as a possible pathway for movement of nitrate from land surface to groundwater[18,20,21]. Prior to 1975, well construction practices in Kansas commonly included gravel packing to the land surface. Implementation of legislation in 1975 required that wells be grouted from land surface to at least 7 m deep.

Irrigation well data was used for this analysis because of the existence of permitted water-right data indicating the age of the wells[5]. The irrigation well data is representative of Areas 1 and 2. These two areas represent both the deep and shallow High Plains aquifer. Correlation of depth and age within these two areas was not significant.

The Mann-Whitney results listed in Table 1 for age of well show a statistically lower nitrate concentration in irrigation wells that were installed after 1975. The age-of-well analysis suggests that well construction practices and the length of time that potentially polluting activities have occurred near a well may have an impact on the occurrence of higher nitrate concentration in older wells.

## Depth of Well

Research in Nebraska has shown that a negative correlation exists between nitrate concentration and depth of well, indicating that shallower wells are more likely to be contaminated than deeper wells[18,21]. Research in Kansas also indicates a weak but significant negative correlation (Table 1). Evaluation of nitrate concentration with well depth within each geographic area also shows a similar weak but significant negative correlation. In addition, the Mann-Whitney test shows that wells shallower than the median depth of 33 m have higher nitrate values than the deeper wells. Of the wells shallower than the median depth, 28% have nitrate values greater than 10 mg/l, as opposed to 13% of the deeper wells.

## CONCLUSIONS

Nitrate contamination of groundwater in Kansas is a current and probable future problem. Even deep aquifer zones show detectable levels of nitrate that are related to farming practices. Nitrate

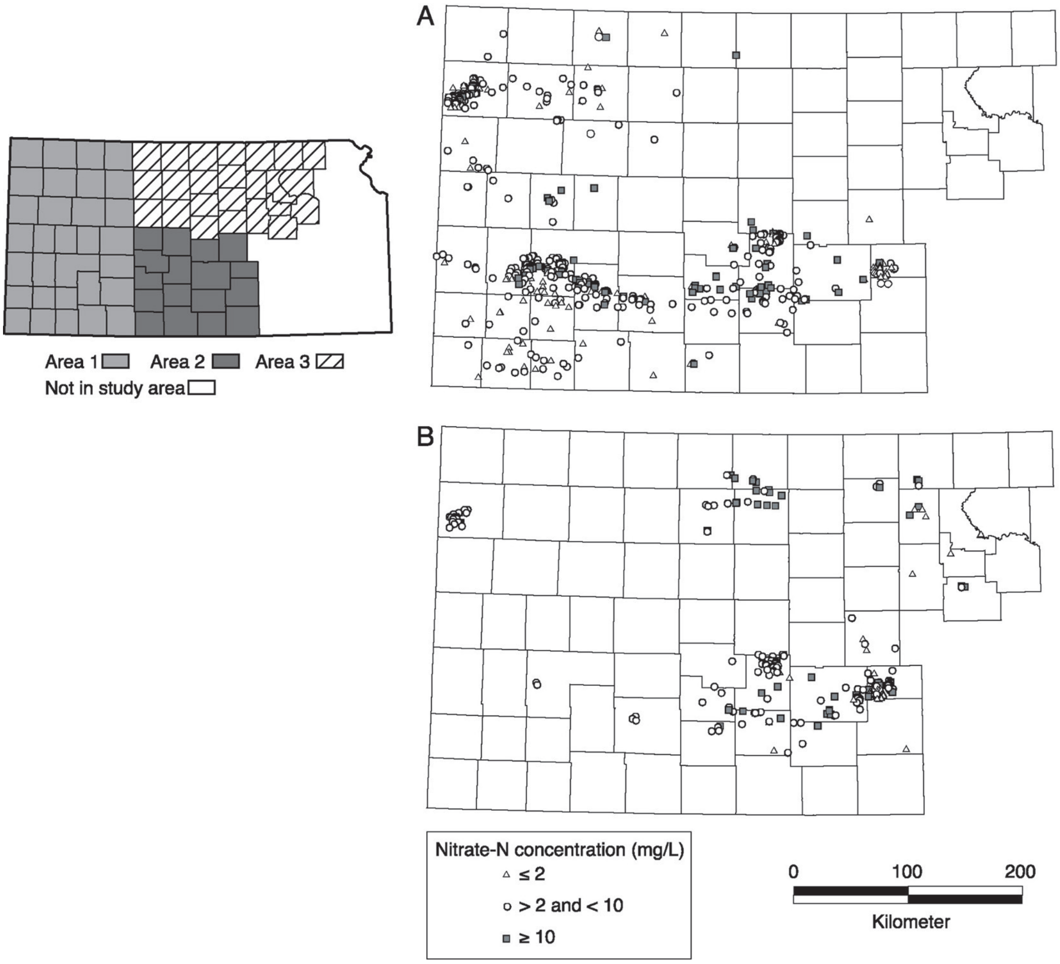

**FIGURE 5.** A: Location of sampled irrigation wells in Kansas. B: Location of sampled nonirrigation wells (domestic, monitoring, and municipal) in Kansas. Index map shows division of state into geographic subunits based primarily on geology, soil type, precipitation, and aquifer depth[6]. Intermittent and dry streams are predominant in Area 1. Perennial streams occur in Areas 2 and 3.

**TABLE 1**
**Summary Table of Significant Statistical Results[6]**

| Factor Evaluated | Number Samples | Median Nitrate-N | Range Nitrate-N |
|---|---|---|---|
| **Geographic area (Fig. 5)** | | | |
| Area 1 (Deep High Plains aquifer) | 358 | 4.3 | 0.02–69.7 |
| Area 2 (High Plains aquifer) | 333 | 5.7 | 0.02–77.4 |
| Area 3 (Alluvial aquifers) | 56 | 7.7 | 0.10–58.4 |
| Kruskall-Wallis *p* value = 0.0339 | | | |
| **Age of irrigation well** | | | |
| ≤ 1975 | 276 | 5.2 | 0.02–43.3 |
| > 1975 | 196 | 4.5 | 0.02–28.7 |
| Mann-Whitney *p* value = 0.0069 | | | |
| **Median depth** | | | |
| < 33 m | 270 | 6.1 | 0.02–61.4 |
| ≥ 33 m | 266 | 4.4 | 0.02–69.7 |
| Mann-Whitney *p* value = 0.0006 | | | |

All nitrate values vs. depth of well: Spearman rho = -0.144 ($p$ = 0.0008).

concentrations vary with geographic location in the state, well depth, and age of well construction. The trend of increasing concentration of nitrate in the groundwater suggests that concentrations will continue to increase under current land-use practices. Nitrogen isotope analyses indicate that past and present fertilizer practices, as well as animal or human waste sources, are major contributors to nitrate contamination of Kansas groundwater. To prevent further contamination, proper management of all nitrate sources is necessary. Best management practices that are recommended throughout Kansas include split applications of fertilizer, preplanting soil testing to determine nitrogen load, use of groundwater nitrate load as part of the fertilizer application, and evaluating water application rates to minimize leaching of excess nitrogen from the fields.

## ACKNOWLEDGMENTS

The authors wish to thank the various agencies and personnel who assisted in the collection and analysis of water samples. The authors also wish to thank Marla and Dana Adkins-Heljeson and Mark Schoneweis for assistance with editing and graphics.

| | | |
|---|---|---|
| Received: | July | 17, 2001 |
| Revised: | October | 11, 2001 |
| Accepted: | October | 29, 2001 |
| Published: | November | 9, 2001 |
